# Characterization of dietary choline uptake by the gut microbiome reveals choline assimilating microbes and influences on host choline metabolism

**DOI:** 10.21203/rs.3.rs-8491198/v1

**Published:** 2026-01-07

**Authors:** Elizabeth Johnson, Paula Bañuelos, Janine Comrie, Annett Richter, Henry Le, Sharon Thompson

**Affiliations:** Cornell University; Cornell University; Cornell University; Cornell University; Cornell University; Cornell University

**Keywords:** gut microbiome, choline, click chemistry, diet-microbiome, nutrition

## Abstract

Choline is an essential nutrient with diverse roles in host metabolism; however, the current understanding of its microbial fate is largely restricted to trimethylamine production. Here, we apply the BioOrthogonal-labeling, Sorting, Sequencing, and mass Spectrometry (BOSSS) workflow to map dietary choline-specific gut microbial interactions. Using an alkyne-modified choline analog (propargylcholine) in mice, we fluorescently label and flow-sort choline-assimilating gut bacteria, identifying a varied set of taxa enriched in probiotic species, including *Limosilactobacillus reuteri* (*L. reuteri*). In vitro studies confirm that *L. reuter*i assimilates choline and converts it to long chain fatty acylcholines. Metabolomic and transcriptomic profiling revealed that *L. reuteri* colonization, with a choline sufficient diet, selectively elevates serum choline and increases lysophosphatidylcholine production, findings corroborated by transcriptomic evidence of upregulated hepatic genes involved in lipid metabolism. These findings uncover a new metabolic fate for dietary choline, expand the known repertoire of microbiome-derived lipids, and illustrate how specific host–microbe–diet interactions can influence host status.

## Introduction

Choline is an essential nutrient for many mammalian processes^1^, and a deficiency in dietary choline can lead to a range of health issues, such as liver disease^2^, muscle atrophy^3,4^, and neurodegenerative disorders^5–7^. One of the most widely recognized contexts for choline supplementation is the prenatal period, with recommendations extending through lactation^8^. As individuals mature, common dietary sources of choline include milk, eggs, broccoli, beans, and white rice. Dietary choline is absorbed in the digestive tract and is utilized for the production of metabolites such as betaine^9^, phosphatidylcholine^10^, lysophosphatidylcholine^11^, and sphingomyelin,^12^ all of which contribute to vital processes such as neurotransmitter synthesis^13^, lipid transport^14^, osmolyte regulation^9^, and one-carbon metabolism^15,16^.

Recent research has highlighted the gut microbiome as a contributing participant in choline metabolism^17^. Certain gut microbes, particularly those harboring the *cutC* gene cluster, convert choline into trimethylamine (TMA)^18,19^, thereby altering the bioavailability and metabolic fate of dietary choline^20^. Despite the impact of these microbes on host outcomes ranging from cardiovascular health^21,22^ to body odor^23^, current dietary guidelines for choline do not account for the microbiome’s quantifiable influence on choline metabolism^17,24^. Since dietary choline consumption and supplementation leads to increased choline availability to the gut microbiome^25^, it is critical to understand the varied ways that gut microbes can contribute to choline absorption and metabolism. Moreover, while microbial conversion of choline to TMA is well-characterized^19,26^, choline is a versatile nutrient, likely entering other gut microbial metabolic pathways. As with host-derived conversions, microbial-specific conversions of choline may lead to previously uncharacterized metabolites that participate in a range of bioactivities, altering the status of host health.

To facilitate the identification of dietary choline-interacting microbes and microbial-dependent dietary choline-derived metabolites, we have utilized our unique workflow that includes **B**io**O**rthogonal-labeling, **S**orting, **S**equencing, and mass **S**pectrometry (BOSSS)^27^ which allows us to identify new diet-microbiome interactions. To explore the fate of choline, we chose to use an alkyne-modified analog of choline, called propagylcholine, which enables bioorthogonal tracing of dietary choline. This choline derivative has been previously characterized as a suitable choline tracer^28,29^. After treating mice with dietary propargylcholine, we isolated gut bacteria and used click chemistry to label gut microbes containing choline-derived molecules with an azide-bearing fluorophore. Microbes that take up diet-derived choline can be sorted by their fluorescence and then sequenced to identify the taxa capable of consuming dietderived choline. The small mass difference between propagylcholine and native choline further allows us to identify microbial transformations of the dietary choline using techniques in comparative metabolomics. This workflow has previously identified novel sphingolipid^27^ and cholesterol^30^ interactions with the gut microbiome. This choline-centric work is the first application of the BOSSS method on a non-lipid dietary input, demonstrating the versatility of this approach.

Here, we identified several probiotic bacteria, including *Limosilactobacillus reuteri* (*L. reuteri*), that interact with dietary choline in mouse models of diet-microbiome interactions. We demonstrated that *L. reuteri* assimilates choline *in vitro* and converts it to various acylcholine species. In gnotobiotic mice, microbe-derived choline metabolites were transferred to host tissues, confirming the transfer of nutrients across host-microbe interactions. In addition, metabolomic and transcriptomic profiling further revealed that *L. reuteri* colonization, in the context of choline sufficiency, selectively elevates choline levels in circulation (serum), cecal content lysophosphatidylcholine species, and upregulates genes involved in lipid metabolism. Together, these findings define a previously unrecognized metabolic fate of dietary choline, broadening the repertoire of known microbe-derived lipids, and illustrate how specific host-microbe interactions can influence host status.

## Results

### Identification of dietary choline-utilizing gut microbes using the BOSSS workflow

**Figure 1:**
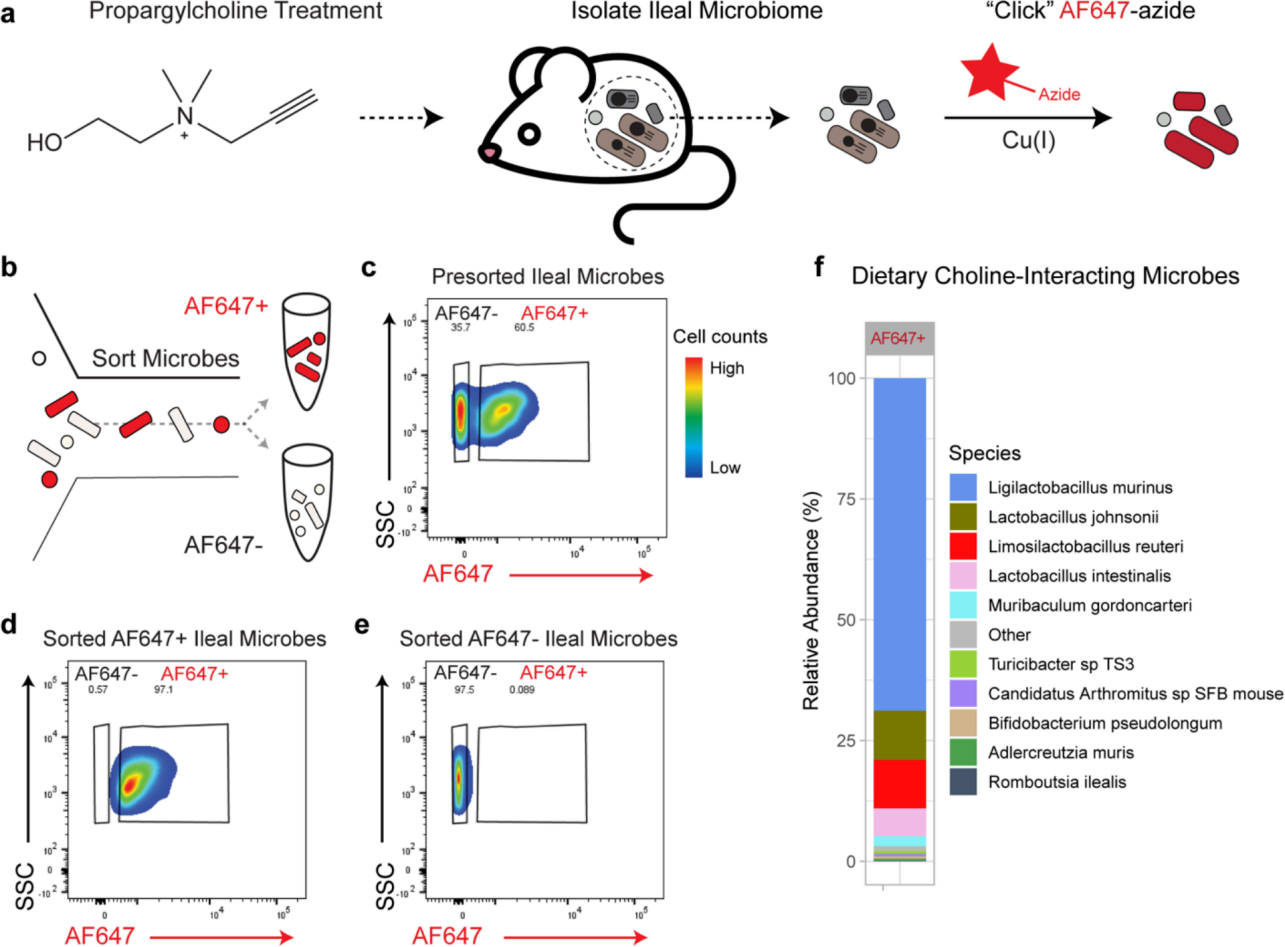
Identification of diet-derived choline-interacting microbes using bio-orthogonal labeling, fluorescence-activated cell sorting (FACS), and shotgun metagenomic sequencing. **(a)** Schematic of the in vivo labeling workflow to identify dietary choline-interacting microbes. Mice (n=4) were orally gavaged with 2 mg of propargylcholine daily for five days, and the ileal contents were collected. Microbial cells were isolated and labelled via cooper-catalyzed azide-propargyl cycloaddition “click chemistry” with AF647-azide to fluorescently tag dietary choline-interacting bacteria. **(b)** Schematic showing the FACS-based separation of microbial populations into dietary choline-interacting (AF647+) and non-interacting (AF647−) populations. **(c)**Flow cytometry pseudocolor plots show AF647 fluorescent intensity (AF647, x-axis) versus side scatter (SSC-A, y-axis) showing labeling of bacteria from mice fed propargylcholine and tagged via click chemistry with AF647-azide. The gate labeled AF647− shows the bacterial population that did not interact with propargylcholine, and the gate labelled AF647+ contains the bacterial population that did uptake of propargylcholine. Plot shown is a pooled ileal sample of four replicates. Flow cytometry plots show the purity of the **(d)** AF647+ and the **(e)**AF647− sorted bacterial populations. **(f)** Stacked bar plot shows the relative abundance of the ileal bacterial species that interact with dietary choline (AF647+) based on shotgun metagenomic sequencing.

To identify choline-interacting microbes, we initiated the BOSSS workflow by orally administering propargylcholine to mice (Fig. 1a). After oral gavage with propargylcholine, ileal contents were collected for downstream analysis. Microbial cells were isolated and subjected to the copper-catalyzed azide–alkyne cycloaddition (“click chemistry”)^29^ with an azide-conjugated fluorophore (AF647-azide) (Fig. 1a) and were sorted via fluorescence activated cell sorting (FACS) (Fig. 1b). Flow cytometry of pooled ileal microbial samples (n = 4) revealed two distinct populations: AF647-positive (AF647+) cells representing choline-interacting bacteria (60.5%), and AF647-negative (AF647−) cells representing non-interactors (35.7%) (Fig. 1c; Supplemental Fig. 1a-d). To isolate the AF647+ population, we performed FACS using a gating strategy detailed in (Supplemental Fig. 1e). To ensure the accuracy of the sort, post-sort flow cytometry was performed on subsamples of each population (Fig. 1d–e). Post-sort analysis showed high purity, with 97.1% of cells in the AF647+ fraction (Fig. 1d) and 97.5% in the AF647− fraction (Fig. 1e) correctly gated, validating the specificity and efficiency of the BOSSS workflow for isolating choline-utilizing microbes *in vivo*.

To determine the taxonomic composition of choline-utilizing bacteria, we extracted genomic DNA from the AF647+ population and performed shotgun metagenomic sequencing. Relative abundance analysis revealed a diverse microbial community, including several taxa with known probiotic potential, including *Ligilactobacillus murinus*, *Lactobacillus johnsonii*, *Limosilactobacillus reuteri* (*L. reuteri*), *Lactobacillus intestinalis*, *Bifidobacterium pseudolongum*, and *Romboutsia ilealis*, among others (Fig. 1f). Indeed, Lactobacilli and Bifidobacteria are known to import choline for osmoregulation^31^. Interestingly, these species lack the TMA-lyase gene (Supplemental Fig. 2), suggesting a fate for choline that diverges from TMA production. We found that *L. reuteri* and *L. johnsonii* were the most abundant bacterial species associated with human health. Both are readily culturable and widely available as commercial probiotics, underscoring their translational relevance. For these reasons we continued our investigations with *L. reuteri*. Together, these findings demonstrate the BOSSS workflow effectively isolates and identifies previously uncharacterized microbes capable of utilizing dietary choline *in vivo*.

### Metabolomics reveals *L. reuteri* assimilates choline and produces acylcholines

**Figure 2:**
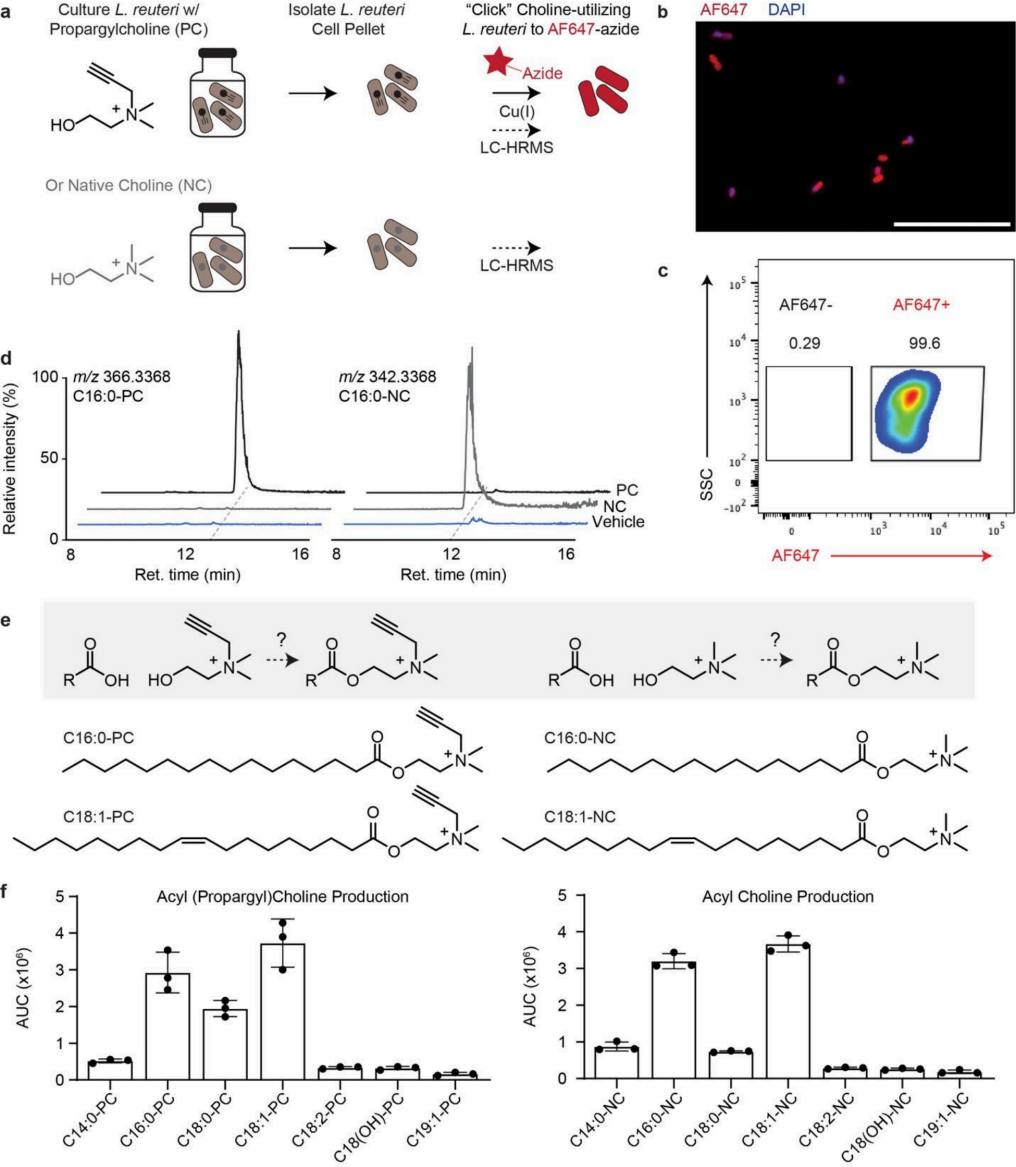
Choline utilization by L. reuteri in vitro. **(a)** Scheme depicting workflow to determine L. reuteri choline utilization in vitro. L. reuteri was cultured in MRS media supplemented with 60 mM of propargylcholine (PC). The bacterial cells were then collected and click-stained with AF647-azide. **(b)** A representative confocal fluorescent microscopy image of L. reuteri cultured in media supplemented with 60 mM of propargylcholine, then isolated and click-stained with an azide-conjugated AF647 detection reagent (red). DNA was stained using a mountant with DAPI (blue). Images were acquired at 100x magnification. Scale bar 20 μm. **(c)** Flow cytometry density plot of L. reutericultured with 60 mM of propargylcholine. The samples were then click-stained with AF647-azide. The x-axis shows the level of AF647 intensity, and the y-axis shows the side scatter (SSC). **(d)** Extracted ion chromatograms from LC-MS analysis showing the detection of C16:0-PC (m/z 366.3368) and the native version, C16:0-NC (m/z 342.3368) in L. reuteri cultured in vitro in MRS media supplemented with either 60 mM of propargylcholine. The x-axis represents retention time (min), and the y-axis represents ion relative intensity. Chromatograms are shown for L. reuteri cultured with 60 mM of propargylcholine (black) and 60 mM of native choline (grey), or no treatment control. Schematic of the proposed pathway for **(e)** Scheme for the production of propargyl- and native-acylcholine. **(f)** Area under the curve (AUC) quantification of various acylcholines from extracted ion chromatograms (EICs) of L. reuteri cultured with 60 mM of propargylcholine or native choline.

To confirm that *L. reuteri* utilizes choline, we cultured *L. reuteri* in de Man, Rogosa, and Sharpe (MRS) media supplemented with 60 mM of propargylcholine or native choline as a control (Fig. 2a). *L. reuteri* assimilation of choline was observed by leveraging the propargyl moiety of propargylcholine and conducting click staining as described above. Fluorescence microscopy revealed bright AF647 (red) and DAPI (blue) signals, indicating uptake of propargylcholine by *L. reuteri* (Fig. 2b). Flow cytometry further showed that 99.6% of cells were AF647^+^ (Fig. 2c), confirming utilization of propargylcholine by *L. reuteri in vitro*. Moreover, we were able to monoassociate mice with *L. reuteri* and confirm the *in vivo* uptake of diet-derived propargylcholine through the detection of AF647 positive *L. reuteri* isolated from ileal contents (Supplemental Fig. 3). From flow cytometry-based analysis of the microbiome-contents, a large proportion (45.9%) of L. reuteri interacted with diet-derived propargylcholine. The distinct difference between interactions may have been limited by the dose

After confirming that *L. reuteri* can uptake choline in isolated cultures, we elected to determine if *L. reuteri* transformed choline to more complex metabolites, as is common with choline. To accomplish this, we performed comparative metabolomics and leveraged the distinct mass shift (+24) introduced by the propargyl moiety in propargylcholine. Following cell pellet isolation, metabolites were extracted and analyzed via LC-MS. Comparative analysis revealed a metabolite with m/z 366.3368 corresponding to an C16:0-PC (propargylpalmitoylcholine), detected exclusively in the propargylcholine-treated *L. reuteri* sample (Fig. 2d–f, Supplemental Fig. 4) and absent in the native choline control (Fig. 2d, Supplemental Fig. 4) and the untreated sample. MS2 analysis and comparison of synthetic standard confirm the production of C16:0-PC (Supplemental Fig. 5), a type of long-chain acylcholine. The native version of palmitoylcholine (C16:0-NC) was also detected in the sample treated with native choline (Fig. 2f), and comparisons with commercial synthetic standard further validate identity. Further analysis revealed the production of C14:0-PC, C18:0-PC, C18:1-PC, C18:2-PC, C18(OH)-PC, and C19:1-PC (Fig. 2f) as well as the native version of these acylcholines in the native choline-treated sample (Fig. 2f). We speculate upon choline assimilation, *L. reuteri* acylates choline, likely with the coenzyme-A thioesters variants of these fatty acids (Fig. 2e). To our knowledge, this is the first identification of a gut dwelling bacteria which produces long-chain acylcholines.

### Metabolic transfer from *L. reuteri* to host tissues

**Figure 3:**
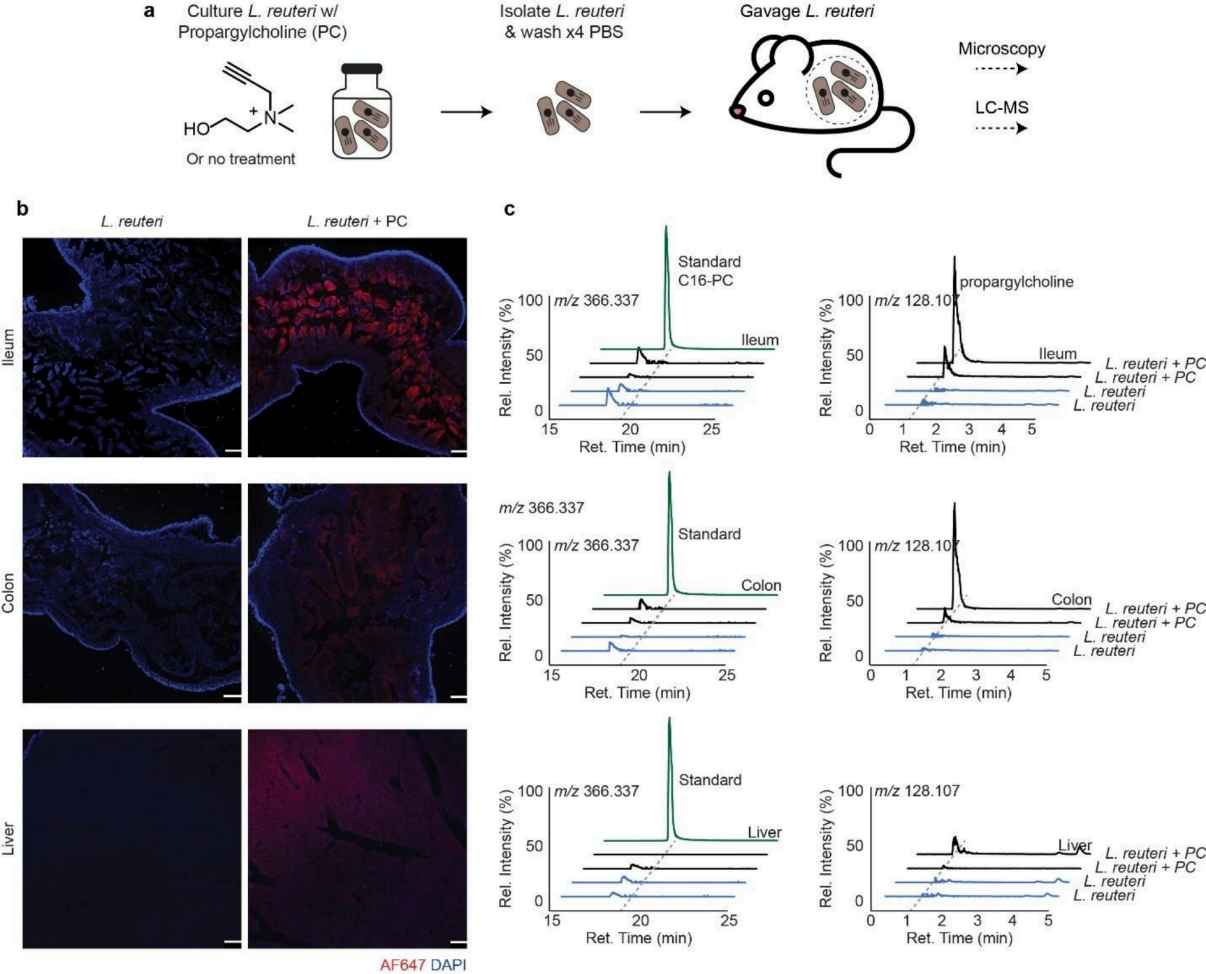
L. reuteri metabolites are detected in host tissues. **(a)** Scheme for determining metabolite transfer. Either PC or vehicle is added to L. reuteri cultures. Bacterial pellets are harvested and wash four times with phosphate buffered saline (PBS). Mice are then gavaged with washed bacterial pellets. **(b)** Fluorescence microscopy images of ileum, colon, and liver tissue sections showing incorporation of L. reuteri-derived propargylcholine metabolites (AF647 signal, red). The right column depicts tissues from mice colonized with L. reuteri grown in media supplemented with propargylcholine; the left column shows tissues from control mice colonized with untreated L. reuteri. Nuclei are counterstained with DAPI (blue). Images were acquired at 5× magnification: scale bar, 200 μm. **(c)** Mice tissues from the bacterial metabolite transfer assay were harvested, extracted, and analyzed via LC-MS. Extracted ion chromatograms depict the lack of detection of C16:0-PC in ileum, colon or liver and the affirmative detection of propargylcholine in ileum, colon, and liver.

To determine whether *L. reuteri* choline-derived metabolites are transferred to the host, we cultured *L. reuteri* with propargylcholine (*L.reuteri*-PC), native choline (*L. reuteri*-NC), or no treatment (*L. reuteri*-NT) (Fig. 3a). After harvesting the live bacterial pellet, the treated *L. reuteri* cells were washed four times with PBS and administered to germ-free mice by oral gavage once daily for three consecutive days. Upon sacrifice and tissue harvest, post-click imaging via fluorescence microscopy revealed host tissue incorporate choline-derived metabolites from *L. reuteri* in the ileum, colon, and liver (Fig. 3b). In the ileum, fluorescence microscopy of cryosectioned tissue with DAPI (blue) and click-enabled AF647-azide (red) demonstrated a high AF647 signal in the villus of mice monoassociated with *L. reuteri*-PC, a signal absent in controls (Fig. 3b), indicating that choline-derived metabolites are incorporated into the ileum. The AF647 signal, although present in the colon, is modest compared to the ileal signal, suggesting the ileum as the main entry site. We also observe modest signal in liver tissue, consistent with dilution of the metabolite(s) as it distributes throughout the body.

We then performed metabolomics analysis of the ileum, colon, and liver to determine what metabolites were transferred. Interestingly, we did not detect the bacterially-derived long-chain acylcholines (Fig. 3c). Comparative analysis of all three systems revealed that the only alkyne-bearing differential feature was propargylcholine (Fig. 3c). The liver showed substantially less propargylcholine, consistent with dilution due to systemic delivery. Although we anticipate host integration of PC into host lipids, individual metabolites may lay below the detection limits of our MS instrumentation. Metabolomic inspection of the bacterial cecal contents showed substantially reduced or absent long-chain acylcholines, suggesting that either the bacteria hydrolyze the acylcholines *in situ* to metabolic transfer, or acylcholines are hydrolyzed upon receipt by host tissues. Overall, this suggest that *L. reuteri* transfers choline to host tissues, either via acylcholines or choline directly, and at some point along the transfer, acylcholines are hydrolyzed.

### Choline and *L. reuteri* in tandem modulate host lipid metabolism

**Figure 4:**
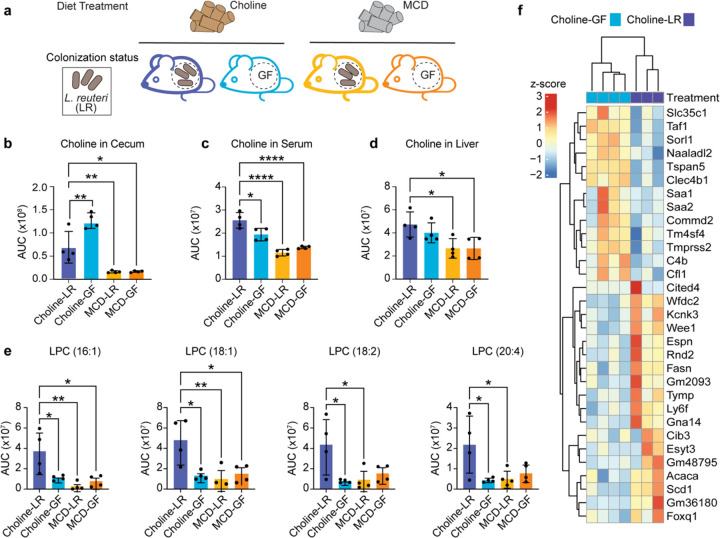
Host metabolic changes driven by dietary choline-L. reuteri interactions. **(a)** Schematic of experimental design. Germ-free mice were kept germ-free (GF) or mono-colonized with L. reuteri (LR) via oral gavage and placed on a choline-sufficient diet (choline) or a methionine choline-deficient diet (MCD) for 3 weeks (n=4). Mice were euthanized, and samples were then analyzed. Area under the curve (AUC) quantification of choline from extracted ion chromatograms (EICs) of **(b)** cecal contents, **(c)** serum, and **(d)**liver tissue. AUC values were obtained from EICs generated by LC-MS monitoring m/z = 104.2 corresponding to choline. **(e)** The abundance (AUC) of lysophosphatidylcholine (LPC) species in the cecal contents of mice. 16:1, 18:2, 20:4, 18:1, and were detected via LC-MS in the cecum of mice. The x-axis represents the treatment conditions, and the y-axis shows the integrated AUC from extracted ion chromatograms, reflecting abundance. Data are presented as mean +/− SEM. Statistical Significance was determined using one-way ANOVA followed by Tukey’s multiple comparison test with an α = 0.05. P-values were adjusted for multiple testing using the built-in Prism multiple-comparison correction. *p < 0.05, **p<0.01, ***p<0.001, ****p<0.0001. **(f)**Heat-map of mice liver RNAseq data depicting relative expression of genes associated with lipid metabolism with and without L. reuteri seeded in germ-free mice fed a choline-rich d

To understand the combined effects of *L. reuteri* and dietary choline on host choline metabolism, we established four experimental groups for comparative metabolomics and transcriptomic analysis. Mice were fed either a choline sufficient diet (choline) or a methionine-choline deficient diet (MCD), and within each dietary condition, mice germ-free mice were either monoassociated with *L. reuteri* (choline-LR or MCD-LR) or maintained germ-free (choline-GF or MDC-GF) (Fig. 4a). This factorial design enables isolated identification of effects dependent on both *L. reuteri* and dietary choline.

As expected, metabolomics revealed that mice fed a MCD diet (MCD-LR and MCD-GF) exhibited significantly lower cecal choline levels compared to those on the choline-sufficient diet (choline-LR and choline-GF). Notably, cecal choline levels were significantly reduced in the choline-LR group relative to the other groups (Fig. 4b). In the liver, choline levels were significantly lower between choline-LR group compared to MCD-LR and MCD-GF. However, hepatic choline levels did not differ significantly between the choline-LR and choline-GF groups (Fig. 4d), suggesting that dietary choline is the primary determinant of hepatic choline availability. In serum, choline concentrations were significantly elevated in the choline-LR group compared to all other groups (choline-GF, MCD-LR, and MCD-GF) (Fig. 4c), suggesting that *L. reuteri* colonization in the context of choline sufficiency may enhance systemic choline bioavailability. With microbial communities capable of producing TMA resulting in lower serum choline levels^24^, this work demonstrates how microbial communities with different choline modifying capacities can have varied and pronounced effects on host choline availability.

In addition, comparative metabolomics on cecal contents across four experimental groups revealed a significant enrichment of four lysophosphatidylcholine (LPC) species, LPC (16:1), LPC (18:2), LPC (20:4), and LPC (18:1) specifically in the choline-LR group (Fig. 4e). LPCs are bioactive phospholipids implicated in diverse physiological processes, including membrane remodeling^32^, cellular signaling^33^, and inflammatory responses^33,34^. Elevated LPCs in the choline-LR group point to a metabolic outcome of host-microbe-diet interactions, highlighting a cooperative effect between *L. reuteri* and choline on host lipid metabolism.

To further investigate the origin of the differential LPCs, we performed RNA sequencing on the livers of the previously mentioned treatment groups. Principal component analysis of variance-stabilized liver transcriptomes, using choline-LR group as the reference condition, revealed clear clustering by dietary treatment (Supplemental Fig. 6a), indicating that dietary choline is the primary factor influencing hepatic gene differentiation. Next, we focused on determining hepatic expression differences between differently colonized mice placed on a choline-sufficient diet. Differential expression analysis identified 31 genes significantly altered between choline-GF (n=4) and choline-LR (n=3) conditions (*p*-value <0.05), with red indicating upregulated genes and blue downregulated genes (Fig. 4f). Hierarchical clustering revealed a separation between the choline-GF and choline-LR-treated mice, with samples treated with choline-LR exhibiting up-regulation in genes related to lipid and fatty acid metabolism. Notably, genes such as stearoyl-CoA desaturase 1 (*Scd1*), acetyl-CoA carboxylase alpha (*Acaca*), and fatty acid synthase (*Fasn*) were significantly upregulated in response to *L. reuteri* mono-colonization, further suggesting that *L. reuteri* modulates host lipid metabolism. Collectively, these findings demonstrate that while dietary choline is a dominant factor shaping choline metabolism, its interaction with *L. reuteri* can significantly modulate choline assimilation, particularly along the digestive track and in host serum.

## Discussion

In this study, we identify dietary choline-interacting microbes, including *L. reuteri*, *L. johnsonii, L. intestinalis, B. pseudolongum, and B. intestinalis,* using BOSSS, a method to track dietary nutrient transfer in host-microbe systems. Among the dietary choline-interacting microbes, we elected to further study *L. reuteri* for its known association with promoting host health^35,36^. Using *in vitro* cultures, we demonstrated that *L. reuteri* assimilates choline and converts it to several distinct acylcholines, including C16:0, C14:0, C18:0, C18:1, C18:2, C18(OH), and C19:1. Gnotobiotic mice studies revealed that *L. reuteri* transfers choline metabolites to the host, as noted in our ileum, colon, and liver images. Comparative metabolomics identifies that choline of gut microbial origin is transferred to host tissue yet identified *L. reuteri*-derived acylcholines do not persist, suggesting acyl hydrolysis. Finally, mice monocolonized with *L. reuteri* and fed a choline-sufficient diet showed increased serum choline and cecal lysophosphatidylcholine levels. These findings suggest that *L. reuteri* uptake of dietary choline alters host choline metabolism.

Our findings highlight the continued utility of the BOSSS workflow as an unbiased strategy for characterizing how gut microbes utilize dietary choline. While propargylcholine, a biorthogonal analog of choline, has been used to investigate choline-associated membrane dynamics in mammalian cells^28,29^ and choline level in mouse tissue^29^, this study represents the first application of propargylcholine within the BOSSS framework to trace direct interactions between dietary choline and gut microbes.

Previous research characterizing choline-microbiome interactions consisted mostly of studies focusing primarily on the production of TMA^17^. Interestingly, the microbes identified in our study do not possess the *cutC* gene necessary for converting choline to TMA, suggesting a previously uncharacterized metabolic interaction. Indeed, the production of acylcholines highlights a novel bacterial transformation and implicated unstudied host biology. Overall, these findings enhance our understanding of the metabolic capabilities of gut microbes and highlight the potential of propargylcholine-BOSSS for uncovering new interactions between choline and microbes.

Although acylcholine production by *L. reuteri* may underlie the observed LPC differences, these hypotheses may require further investigation. Indeed, acylcholines have known signaling functions, many of which involve acetylcholine, a well-characterized neurotransmitter.^37^ However, the long-chain acylcholines, particularly myristoylcholine^38^, are far less characterized and may affect host systems^39^. Unlike acetylcholine, long-chain acylcholines have longer circulating half-lives and have been shown to modulate cholinergic systems, influencing a wide range of processes, including movement, autonomic function, cognition, immune responses, and others^39–41^.

Although our work focused on *L. reuteri*, our BOSSS pipeline identified a broader community of interacting microbes. Future studies examining other choline-interactors will be important to defining how other taxa contribute, and how the collective bacterial community functions to shape host metabolic responses. Additionally, although we confirm the production of acylcholines, the precise fate of these metabolites is not yet fully characterized. This study, along with others, can facilitate the identification of additional choline-microbe-interactions that advance our understanding of how dietary choline influences host systems, and solicits personalized nutritional strategies that account for microbiome-driven variability in choline metabolism.

## Methods

### Animal experiments.

All mouse experiments were performed according to a protocol approved by the Cornell University Institutional Animal Care and Use Committee (protocol no. 2010–0065).

### In vivo dietary choline uptake.

Female, 9-week-old, conventionally raised, murine pathogen-free Swiss Webster mice (n=4) were purchased from Taconic Bioscience. The mice were administered 2 mg of propargylcholine dissolved in 100 μL of phosphate-buffered saline (PBS) via oral gavage once daily for five days. They were housed four per cage in a climate-controlled environment with a 12-hour light/dark cycle and were provided with a breeder diet (LabDiet 5021) with ad libitum access to autoclaved water.

On the fifth day, the mice received the final gavage and were then fasted for 3 hours before euthanasia by decapitation. Intestinal contents and tissues were carefully collected and snap-frozen using liquid nitrogen to preserve their integrity. The samples were subsequently stored at −80 °C to ensure long-term stability for future analysis and processing.

### Isolation and fixation of microbial cells from intestinal content samples.

The procedure was adapted from the workflow described in Lee et al.^1^ Intestinal contents were removed from −80°C and allowed to thaw at room temperature for 2 to 3 minutes. Next, 1 mL of sterile PBS was added directly to the sample tube. The samples were then vortexed until homogenized and sonicated for 10 seconds, alternating between 1 second on pulse and 2 seconds off pulse at an amplitude of 1, using a Qsonica Ultrasonic Processor (model Q700 with a water bath adaptor, model 431C2) to separate the microbiome from debris. After sonication, the samples were allowed to settle at room temperature for 2 minutes and then centrifuged at 200 g for 30 seconds. The supernatant was filtered into a 5 mL round-bottom polystyrene test tube using a 35 μm cell strainer snap cap (Falcon, 352235). The supernatant was transferred to a new 1.5 mL microcentrifuge tube (VWR, 1615–5500) and centrifuged for 5 minutes at 8,000 g. The bacterial pellets were washed twice with 1% bovine serum albumin (BSA) in PBS. After discarding the supernatants, the bacterial cell pellet was resuspended in a 10% formalin solution for 10 minutes. The bacterial cells were then washed again with 1% BSA/PBS. For permeabilization, 0.1% Triton X-100 in PBS was added and incubated for 10 minutes at room temperature before a final wash with 1% BSA/PBS.

### Cu(I)-catalyzed azide-alkyne cycloaddition (click) staining.

Bacterial and tissue samples containing propargylcholine were fluorescently labeled by using Cu(I)-catalyzed azide-alkyne cycloaddition (CuAAC). Click reactions were performed with the Click-&-Go^™^ CuAAC reaction buffer kit (formerly: Click Chemistry Tools, Scottsdale, AZ – now Vector Labs, Newark, CA) according to the manufacturer’s instructions. The reaction mixture consisted of reaction buffer, copper(II) sulfate, a reducing agent and 5 μM of Alexa Fluor 647 azide (AF647-azide) (ThermoFisher, Cat# A10277). For the bacterial samples, 500 μL of the reaction cocktail was added to each permeabilized cell pellet, mixed gently by pipetting, and incubated 30 minutes on a tube rotator (VWR, Cat# 10136–084) at room temperature while protected from light. Following incubation, the cells were centrifuged for 5 min at 8,000 × g and washed five times with 1% BSA/PBS.For mouse tissue sections, 200 uL of the rection cocktail was applied directly to each slide.

Slides were covered with foil and incubated for 30 min at room temperature in the dark. After incubation the reaction solution was removed, and tissue sections were washed three times with 1% BSA/PBS. Slides were then mounted using SlowFade^™^ Diamond Antifade Mountant (ThermoFisher, Cat# S36967) before imaging. Slides were imaged using a Zeiss LSM 710 Confocal or a Leica DM500 fluorescence microscope (Leica, Buffalo Grove, IL). Images were analyzed using Fiji ImageJ software.

### Fluorescence-activated cell sorting to identify propargyl choline-utilizing bacteria.

The general procedure was adapted from the BOSSS workflow demonstrated in Lee et al^1^. To isolate propargylcholine using bacteria, a BD Biosciences FACSMelody^™^ Cell Sorter (BD Biosciences, San Jose, CA) was used. Samples were filtered using a 5 mL round-bottom polystyrene test tube with a 35 μm cell strainer snap cap (Falcon, 352235). The AF647-azide dye was excited using a 640 nm red laser, and fluorescence was captured with a 660 nm/20 nm filter. The gating strategy to focus on the choline-utilizing bacteria population included using the Side Scatter Area (SSC-A) and Forward Scatter Area (FSC-A) to distinguish microbes from debris. We then used the Side Scatter Width (SSC-W) and Side Scatter Height (SSC-H) followed by the Forward Scatter Width (FSC-W) and Forward Scatter Height (FSC-H) parameters, to distinguish single cells from doublets and aggregates. The SSC-A and AF647 parameters were employed to identify and gate the two populations of interest: the choline-utilizing gut microbe population (AF647+) and the non-choline-utilizing microbe population (AF647-). *B. theta* treated with palmitic acid-alkyne (PAA), referred to as *B. theta*^PAA^ was used as a control to establish the positive gate for AF647-positive choline-utilizing microbes. A no-stain sample of *B. theta* and a vehicle (PBS) control ileum sample were used to set the negative gate for AF647 to identify non-choline-interacting microbes. The microbes were then sorted into two different tubes.

### Synthesis of acyl cholines.

1 mL of dry acetonitrile (Fisher Scientific, Cat# AC364311000) and 5 mg of propargyl bromide (Cayman Chemicals, Cat# 25870) were added to a 20 mL glass scintillation vial, followed by the stepwise addition of 150 μL triethylamine (Sigma Aldrich, Cat# 471283) and 16.8 μL of 100 mM DMAP (dissolved in dry acetonitrile) (Sigma Aldrich, Cat# 107700). The solution was chilled over wet ice for 10 minutes, and then 8 μL of palmitoyl chloride (Sigma Aldrich, Cat# P78–100ML) or oleoyl chloride (Sigma Aldrich, Cat# 36850–100ML) was added. The reaction was allowed to proceed over ice for 1 hour, then moved to room temperature (22°C) for another 2 hours. 10 μL of methanol was added and the solution was dried on a speedvac concentrator. The crude material was sonicated into 200 μL methanol, centrifuged at 20,000 × g for 20 min at 4 °C, and the clarified methanolic extract was moved to a fresh vial and diluted 200:1 in an LC vial with methanol before LC-MS analysis.

### DNA extraction.

The sorted bacterial pellet was transferred into a sterile 1.5 mL Eppendorf tube. The pellet was then resuspended in 475 mL TE buffer and 1.5 mL of ReadyLyse lysozyme and incubated at 37°C for 1 hour. Then, 25 mL of 10% SDS and 5.4 mL of proteinase K (NEB) were added and incubated at 55°C for 10 minutes. The tube was then incubated on ice for 5 minutes to stop the reaction, spun down, and transferred into screw-top tubes prefilled with 0.1 mm beads, and 65 mL of 5M NaCl was added. The bead-filled tubes were vortexed vigorously for 30 seconds and incubated on ice for 3 minutes. Then, 500 mL of phenol: chloroform: isoamyl-alcohol (25: 24: 1) (PCl), pH 8.0, was added, and the tubes were gently inverted 10 times. The samples were centrifuged at maximum speed for 10 minutes, and the aqueous phase was transferred into a new tube. The same volume taken from the aqueous phase was added to chloroform: isoamyl alcohol (24:1) into the tube and gently inverted 10 times. The samples were centrifuged at max speed for 10 minutes, then the aqueous phase was transferred into a new Eppendorf tube. 0.1 vol 3M sodium acetate, 1 vol ice-cold isopropanol, and 1 mL glycogen were added into the tube and incubated at −20°C overnight for DNA precipitation. The samples were centrifuged at 4°C for 30 minutes at max speed. The supernatant was discarded and washed 3 times with ice-cold 70% EtOH; the pellet was air-dried and resuspended in DNase-free water.

### Shotgun microbiome sequencing.

The bacterial DNA was extracted as described above. Shotgun metagenomic sequencing library were prepared using the Nextera XT DNA Library Prep Kit (Illumina) with the Nextera XT Index Kit v2 (Illumina) for sample indexing according to manufacturer’s protocol. Sequencing was performed on an Illumina NextSeq 1000 platform using a P2 100-cycle kit, generating unpaired 75 bp reads. Raw sequencing reads were quality filtered using Kneaddata v0.12.0^2^ with default parameters to remove low-quality reads and potential contaminants. Taxonomic classification was performed using MetaPhlAn v4.1.1^3^, optimized for 75 bp reads, with the --*ignore_eukaryotes* and --*ignore_archaea* options to exclude non-bacterial taxa from classification. Post-classification, taxa assigned as “Archaea,” “Viruses,” or “Eukaryota” were removed in R. Additionally, taxa classified as “Unclassified” or ambiguous were excluded. The relative abundance of remaining taxa was scaled to 100 at the Species rank, with excluded taxa removed prior to downstream taxonomic summary visualization. Visualizations were performed using custom plot functions developed in R Studio using ggplot2^4^.

### Percent identity heatmap generation.

The *cutC* gene sequence from *Desulfovibrio desulfuricans* strain L4 was obtained from the NCBI Nucleotide database. The reference genomic region corresponding to the annotated *cutC* locus (accession NZ_CP072608.1:2239761–2242319) was downloaded in FASTA format and used as the query sequence for downstream analyses. To identify homologous *cutC* genes across the identified choline utilizing bacteria and known cutC gene carriers (*Desulfovibrio desulfuricans* (taxid:876), *Klebsiella pasteurii* (taxid:2587529), *Escherichia fergusonii* (taxid:564)), the reference sequence was submitted to the NCBI BLASTn^5^ tool (https://blast.ncbi.nlm.nih.gov/Blast.cgi). The query search was aligned against standard databases (nt etc.) with the organism filter set to *Limosilactobacillus reuteri* (taxid:1598), *Lactobacillus johnsonii* (taxid:33959), *Lactobacillus intestinalis* (taxid:151781), *Ligilactobacillus murinus* (taxid:1622), *Muribaculum gordoncarteri* (taxid:2530390), *Turicibacter* (taxid:191303), *Candidatus Arthromitus* sp. SFB-mouse (taxid:49118), *Bifidobacterium pseudolongum* (taxid:1694), *Adlercreutzia muris* (taxid:1796610), *Desulfovibrio desulfuricans* ATCC 27774 (taxid:525146), *Desulfovibrio desulfuricans* (taxid:876), *Klebsiella pasteurii* (taxid:2587529), *Escherichia fergusonii* (taxid:564), *Limosilactobacillus reuteri* subsp. reuteri JCM 1112 (taxid:557433), *Limosilactobacillus reuteri* 1063 (taxid:1273150), *Limosilactobacillus reuteri* mlc3 (taxid:863369), *Limosilactobacillus reuteri* I5007 (taxid:1340495), *Limosilactobacillus reuteri* SD2112 (taxid:491077), *Limosilactobacillus reuteri* CF48–3A (taxid:525341), *Limosilactobacillus reuteri* MM2–3 (taxid:585517). BLAST search was performed using the program selection of somewhat similar sequences (blastn) and expected threshold of 0.0001. All other values were left as default settings. For each microbial hit, the percentage identity value reported by BLAST was recorded. Percent identity values from all species identified in BLAST output were compiled into a spreadsheet listing each microbe and its corresponding *cutC* percent identity. These data were then imported into R (v4.3.0) for visualization. The heatmap was generated using ggplot2. Briefly, the dataset was read using the readxl package, and the percent identity column was converted to numeric format. Microbial species were ordered by increasing similarity to the reference sequence. A single gene heatmap (representing *cutC*) was plotted using geom_tile(), with a custom color gradient ranging from blue (low percent identity) to red (high percent identity). The final heatmap figure was exported as a vector-based PDF using the ggsave() function.

### *L. reuteri* cultured with propargyl choline.

A glycerol stock of *L. reuteri* ATCC 23272 was cultivated overnight in MRS broth media (Millipore, 69966) at 37°C under anaerobic conditions. Following this, *L. reuteri* OD_600_ was adjusted to an optical density of 0.5. Subsequently, 20 mL of MRS media supplemented with 60 mM of propargylcholine was inoculated with 0.5 mL of the overnight culture of *L. reuteri* and incubated overnight at 37°C under anaerobic conditions. For LC-MS detection of propargylcholine utilization, *L. reuteri* was cultured in 20 mL of MRS media supplemented with either 60 mM of propargylcholine or 60 mM of native choline (choline bromide) (Thermofisher, CAT# C032825G).

### Isolation and fixation of *L. reuteri* cultures.

After the incubation described above, the *L. reuteri* culture was transferred to a 50 mL centrifuge tube (VWR, 89039–660) and centrifuged for 5 minutes at 8,000 g. The cell pellet was then resuspended in 1 mL of PBS and transferred to a new 1.5 mL microcentrifuge tube (VWR, 1615–5500), followed by another round of centrifugation for 5 minutes at 8,000 × g. The bacterial pellets were washed twice with 1% BSA in PBS. The supernatants were discarded, and the bacterial cell pellet was resuspended in a 10% formalin solution for 10 minutes. The bacterial cells were subsequently washed with 1% BSA/PBS, and 0.1% Triton X-100 in PBS was added to permeabilize the cells for 10 minutes at room temperature. Finally, the bacterial cells were washed again with 1% BSA/PBS.

### Fluorescence microscopy.

The bacterial cell suspension was smeared onto glass slides with 10 μL of SlowFade Diamond Antifade Mountant with DAPI (Invitrogen^™^, S36967). Slides were imaged using a Zeiss LSM 710 Confocal or a Leica DM500 fluorescence microscope (Leica, Buffalo Grove, IL). Images were analyzed using Fiji ImageJ software.

### *L. reuteri* cell pellet extraction for metabolomic analysis.

*L. reuteri* cultures were harvested, washed with PBS, and pelleted by centrifugation. To normalize sample input for metabolomic extraction, bacterial biomass was quantified by flow cytometry. Each sample’s event rate (events/sec) was measured using a BD FACS Melody flow cytometer. The sample with the lowest event rate was designated as the reference sample. The normalized solvent volume to add to the cell pellets for extraction was calculated using the formula: Normalized extraction volume (μL) = (Reference sample event rate/Sample event rate) × Reference extraction volume. This approach ensured that an equivalent number of bacterial cells were extracted across all samples for downstream metabolomic analysis. Following normalization, lipids were extracted overnight in methanol. The samples were then dried using a SpeedVac concentrator. The dried metabolome was reconstituted in 1000 μL of methanol, sonicated, and centrifuged to remove particulates. Finally, 80 μL of the supernatant was transferred to LC-MS vials for analysis.

### Gnotobiotic husbandry.

Experimental diets were sterilized by irradiation (50 kGy) and packed in small bags of 1kg to ensure continued sterility. Four-week-old germ-free Swiss Webster mice were purchased from Taconic Bioscience, aseptically transferred to sterile IsoCage P positive pressure cages (Techniplast, West Chester, PA) and housed in an IsoCage positive pressure rack (Techniplast, West Chester, PA). Germ-free mice were put on the experimental diet the same day as *L. reuteri* colonization. The sterility of germ-free animals was assessed by incubating fecal pellets under aerobic and anaerobic conditions on BHIS plates.

### *L. reuteri* metabolite transfer to host experiment.

*L. reuteri* was cultured anaerobically in MRS media overnight at 37°C. The following day, the culture was adjusted to an optical density of 0.5 at 600 nm (OD_600_). A 100 μL aliquot of the bacterial pellet was then inoculated into 20 mL of MRS media, supplemented with either 60 mM propargylcholine, 60 mM native choline, or left untreated as a control. After incubation overnight, the bacterial cell pellets were washed four times with 20 mL of pre-warmed 1X PBS. The culture was again adjusted to an optical density of 0.5 at 600 nm (OD_600_) and centrifuged for 5 minutes at 8,000 × g. The bacterial cell pellets were resuspended in 1 mL of pre-warmed 1X PBS. Germ-free mice were treated with 200 μL of the corresponding bacterial preparation: *L. reuteri* with propargylcholine (n=4), *L. reuteri* with native choline (n=3), or *L. reuteri* without treatment (n=2). The mice were gavaged once a day for 3 days. On the third day, the mice were fasted for 3 hours after the final gavage and then sacrificed via decapitation.

### Preparation of *L. reuteri* for mouse monocolonization.

*L. reuteri* was cultured anaerobically in MRS media overnight at 37°C, after which the culture was adjusted to an optical density of 0.5 at 600 nm (OD_600_). Bacterial pellets were then washed twice with pre-warmed sterile PBS. Four-week-old germ-free mice were administered 100 μL of *L. reuteri* suspended in PBS via oral gavage using a 20-gauge gavage needle (Fine Science Tools). To verify the colonization of *L. reuteri*, fecal pellets were collected and incubated in MRS media overnight at 37°C after one week.

### Choline diet intervention in germ-free and *L. reuteri* monoassociated mice.

Female, 4-week-old, germ-free Swiss Webster mice were randomly assigned to one of four treatment groups. The groups included mice that were fed a choline-sufficient diet (Dyets, 519595) and monoassociated with *L. reuteri* (ATCC, 23272), mice that were fed a choline-sufficient diet and were maintained on a germ-free colonization status, mice that were fed a methionine and choline-deficient diet (MCD) (Dyets, 518810) and monoassociated with *L. reuteri,* and mice that were fed an MCD diet and maintained on a germ-free colonization status. Mice were housed using an IsoCage P setup (described above) with four mice per cage in a climate-controlled environment with 12-hour light and dark cycles with ad libitum access to autoclaved water. After 3 weeks, mice were fasted for 3 hours and were euthanized via decapitation. Intestinal contents and tissues were collected, snap-frozen using liquid nitrogen, and stored at −80°C until further processing.

### Propargylcholine uptake by *L. reuteri* in germ-free mice.

Germ-free mice were monoassociated with *L. reuteri* before proapargylcholine intervention. For propargylcholine-treated mice, mice on the MCD diet were given 1% of propargylcholine in their drinking water for three days. The mice were then fasted for 3 hours and euthanized via decapitation. Intestinal contents and tissues were collected, snap-frozen using liquid nitrogen, and stored at −80°C until further processing.

### Isolation and fixation of *L. reuteri* from intestinal content samples.

The procedure was modified from the workflow demonstrated in Lee et al.^1^ Intestinal contents were removed from −80°C and allowed to thaw at room temperature for 2–3 minutes. Following this, 1 mL of sterile PBS was added directly into the sample tube. The samples were then vortexed until homogenized and sonicated for 10 seconds, using an alternating pattern of 1 second on and 2 seconds off at an amplitude of 1 (Qsonica Ultrasonic Processor, model Q700, with a water bath adaptor, model 431C2) to separate the microbiome from debris. After sonication, the samples were allowed to settle at room temperature for 2 minutes and centrifuged at 200 g for 30 seconds. The resulting supernatant was filtered into a 5 mL round-bottom polystyrene test tube using a 35 μm cell strainer snap cap (Falcon, 352235). The filtered supernatant was then transferred to a new 1.5 mL microcentrifuge tube (VWR, 1615–5500) and centrifuged for 5 minutes at 8,000 g. The bacterial pellets were washed twice with 1% BSA in PBS. After washing, the supernatants were discarded, and the bacterial cell pellet was collected.

### Cryosection.

Mouse tissues were harvested after euthanasia and briefly rinsed with PBS to remove residual blood. The tissues were then embedded in optimal temperature cutting (OTC) compound (Tissue-Tek, 4583) within cryomolds and rapidly frozen using liquid nitrogen-cooled isopentane. Embedded samples were stored at −80°C until sectioning. For cryosectioning, frozen tissue blocks were equilibrated to −20°C in a cryostat chamber (Leica) for 10 minutes before sectioning. Tissues were sectioned at a 10 μm thickness and collected on Superfrost Plus microscopy slides. The slides were fixed in 10% formalin for 10 minutes, rinsed in PBS, and processed for click staining as described above in “Cu(I)-catalyzed azide-alkyne cycloaddition (click) staining” section.

### Liver histology.

Liver samples were harvested, stored in 10% formalin, and held at 4°C for 24 hours. They were placed in cassettes and then transferred to 70% ethanol. The cassettes were submitted to the Cornell Animal Health Diagnostic Center (AHDC) for sample processing and H&E staining.

### RNA sequencing and analysis.

Total liver RNA was extracted using TRIzol reagent (Invitrogen, Cat. No. 15596026) according to the manufacturer’s instructions. Isolated RNA samples were submitted to the Cornell Institute of Biotechnology Genomics Facility for RNA integrity quality control and sequencing.RNA libraries were prepared using the QuantSeq 3’ mRNA-Seq Library Prep Kit FWD for Illumina (Lexogen, Vienna, Austria) following the manufacturer’s protocol. Sequencing was performed on the Illumina NextSeq500 platform, generating 75 bp single-end reads. Raw sequencing reads underwent initial quality assessment using FastQC (v0.12.1).^6^ Adapter trimming and removal of low-quality bases were performed using Trimmomatic (v0.39)^7^ with the parameters SLIDINGWINDOW:4:20 MINLEN:35. The *Mus musculus* reference genome (GCF_000001635.27, GRCm39)^8^ was indexed using STAR (v2.7.10b)^9^ with the options --sjdbOverhang 100 --sjdbGTFtagExonParentTranscript Parent. Trimmed reads were then aligned to the indexed genome using STAR, generating BAM files sorted by coordinate (--outSAMtype BAM SortedByCoordinate). Aligned reads were quantified using HTSeq (v2.0.2)^10^ with htseq-count and the options -s no -r pos -t exon -i pacid -f bam to produce count matrices for downstream differential gene expression analysis.

### Differential Expression.

Differential expression analysis was conducted using DESeq2^11^ in R Studio. A DESeqDataSet was created from the count matrix and associated sample metadata, specifying the experimental condition as the design formula (~ condition). The reference level for the condition variable was set to either “CGF”, “CDGF”, “CLR”, or “CDLR” using relevel (). Differential expression analysis was carried out using the DESeq () function.

Results were extracted using the results () function, and log-fold change shrinkage was applied using the apeglm method through lfcShrink(). Differentially expressed genes were ranked by p-value, and significant genes were filtered using an adjusted p-value threshold of < 0.05. To annotate the results, Ensembl gene IDs were mapped to gene symbols using the biomaRt package,^12^ with annotations retrieved via the useEnsembl () function for *Mus musculus*^13^.

### Sample preparation of cecal and liver samples for metabolomics.

Frozen cecal and liver samples were lyophilized overnight to remove water content. A dry weight-based normalization approach was used to normalize metabolite extraction across biological samples. The dry weights of all samples were recorded, and the sample with the lowest weight was designated as the reference sample. A fixed volume of 1500 μL of extraction solvent (methanol) was added to all samples, regardless of weight. Homogenization was performed to ensure complete solubilization of cecal contents. To account for differences in sample mass, a normalized volume of homogenate to extract was calculated based on the reference sample’s dry weight using the formula: Normalized volume (μL) = (Reference sample dry weight×1500) / (Sample dry weight). Lipids were extracted overnight and transferred to the SpeedVac concentrator. After drying in the SpeedVac, samples were reconstituted in 100 μL of methanol, sonicated, centrifuged, and 80 μL of the supernatant was transferred to LC-MS vials for analysis.

### Sample preparation of serum samples for metabolomics.

Serum samples were normalized by volume prior to extraction. A fixed 100 μL of each sample was aliquoted into microcentrifuge tubes, flash-frozen in liquid nitrogen, and lyophilized overnight. The dried serum metabolome was reconstituted in 100 μL of methanol, sonicated, and lipids extracted overnight. Samples were transferred to the SpeedVac concentrator to remove solvent. The dry metabolome was then reconstituted in 50 μL of methanol, sonicated, centrifuged, and 30 μL of the supernatant was transferred to LC-MS vials for analysis.

### Metabolomics Analysis.

Samples were analyzed using a Thermo Scientific Vanquish Horizon UHPLC System, which was coupled with a Thermo Scientific Q Exactive mass spectrometer for both MS1 and MS2 analyses. MS2 analysis of mouse samples was conducted using a Thermo Scientific Vanquish Horizon UHPLC System that was coupled with a Thermo Scientific Q Exactive HF Orbitrap mass spectrometer. MS1 analysis of mouse samples was conducted using a Thermo Scientific UltiMate 3000 HPLC System, which was coupled with a Q Exactive mass spectrometer. A Kinetex EVO C18 column (150 mm × 2.1 mm, 1.7 μm, Phenomenex, part number 00F-4726-AN) was used for liquid chromatography at 40°C. Solvent A consisted of 0.1% formic acid in water, while solvent B was composed of 0.1% formic acid in acetonitrile. The LC-MS method, which lasted for 32 minutes, utilized an A/B gradient at a flow rate of 0.5 mL/min. The gradient began with 10% solvent B for 2 minutes, then increased linearly to 100% solvent B over a period of 20 minutes. This concentration was maintained at 100% solvent B for an additional 8 minutes. After that, the percentage of solvent B decreased linearly back to 10% by 28.1 minutes and was held at 10% until the end of the 32-minute run. For the 50-minute LC-MS method, the A/B gradient was set at a flow rate of 0.3 mL/min. The gradient started at 10% B for the first 2 minutes, then increased linearly to 100% B by 32 minutes. It was held at 100% B for 16 minutes and then decreased linearly back to 10% B by 48.1 minutes. The system was maintained at 10% B until the end of the 50 minutes. The parameters for the mass spectrometer used in the MS1 analyses were as follows: positive mode, HESI source, spray voltage set to 3.5 kV, capillary temperature at 380°C, and probe heater temperature at 400°C. The sheath gas, auxiliary gas, and spare gas were set to 60, 20, and 1, respectively. The S-lens RF level was adjusted to 50. The resolution was set to 140,000 at m/z 200, with an AGC target of 3e+06 and a maximum ion injection time of 200 ms. The m/z scan range was set from 100 to 1000. Tandem mass spectrometry was conducted using a selected features inclusion list with the Parallel Reaction Monitoring (PRM) method. The same parameters were applied as before, with the following additional adjustments: a resolution of 60,000 at m/z 200, an automatic gain control (AGC) target of 5e+05, a maximum ion injection time of 80 ms, an isolation window of 0.7 m/z, and stepped normalized collision energies of 25, 35, and 45. The Q Exactive was calibrated using Pierce calibration solutions from Thermo Fisher Scientific. Raw data files were converted to MZML files through the ProteoWizard MSConvert GUI. For an untargeted metabolomics analysis of metabolites, peak detection and integration were conducted on MZML files using MZmine (version 4.1.0). The aligned feature list exported from MZmine was imported into R to perform statistical analyses aimed at identifying choline-derived metabolites and those enriched in choline-sufficient mice colonized by *L. reuteri*. To validate the differences in peak areas, targeted metabolomics analysis was performed, and peaks were re-integrated using Skyline.

## Supplementary Files

This is a list of supplementary files associated with this preprint. Click to download.

- BanuelosSI.docx

## Data Availability

All sequencing data are available on the NCBI Sequence Read Archive under Bioproject SUB15425576. All metabolomics data are available on the MassIVE with the accession number MSV000097714.
